# Improving computerized decision support system interventions: a qualitative study combining the theoretical domains framework with the GUIDES Checklist

**DOI:** 10.1186/s12911-023-02273-6

**Published:** 2023-10-18

**Authors:** Janet Yamada, Andrew Kouri, Sarah Nicole Simard, Jeffrey Lam Shin Cheung, Stephanie Segovia, Samir Gupta

**Affiliations:** 1 Daphne Cockwell School of Nursing, Faculty of Community Services, Toronto Metropolitan University, 350 Victoria Street, Toronto, ON M5B 2K3 Canada; 2 Division of Respirology, Department of Medicine, Women’s College Hospital, 76 Grenville Street, Toronto, ON M5S 1B2 Canada; 3 Keenan Research Center, Li Ka Shing Knowledge Institute, St. Michael’s Hospital, Unity Health Toronto, 30 Bond Street, Toronto, ON M5B 1W8 Canada; 4 Division of Respirology, Department of Medicine, University of Toronto, Keenan Research Centre, Li Ka Shing Knowledge Institute, St. Michael’s Hospital, Unity Health Toronto, 30 Bond Street, M5B 1W8 Toronto, ON Canada

**Keywords:** Clinical decision support system, Barriers, Enablers, Uptake, Theoretical domains framework, GUIDES Checklist

## Abstract

**Background:**

Computerized clinical decision support systems (CDSSs) can improve care by bridging knowledge to practice gaps. However, the real-world uptake of such systems in health care settings has been suboptimal. We sought to: (1) use the Theoretical Domains Framework (TDF) to identify determinants (barriers/enablers) of uptake of the Electronic Asthma Management System (eAMS) CDSS; (2) match identified TDF belief statements to elements in the Guideline Implementation with Decision Support (GUIDES) Checklist; and (3) explore the relationship between the TDF and GUIDES frameworks and the usefulness of this sequential approach for identifying opportunities to improve CDSS uptake.

**Methods:**

In Phase 1, we conducted semistructured interviews with primary care physicians in Toronto, Canada regarding the uptake of the eAMS CDSS. Using content analysis, two coders independently analyzed interview transcripts guided by the TDF to generate themes representing barriers and enablers to CDSS uptake. In Phase 2, the same reviewers independently mapped each belief statement to a GUIDES domain and factor. We calculated the proportion of TDF belief statements that linked to each GUIDES domain and the proportion of TDF domains that linked to GUIDES factors (and vice-versa) and domains.

**Results:**

We interviewed 10 participants before data saturation. In Phase 1, we identified 53 belief statements covering 12 TDF domains; 18 (34.0%) were barriers, and 35 (66.0%) were enablers. In Phase 2, 41 statements (77.4%) linked to at least one GUIDES factor, while 12 (22.6%) did not link to any specific factor. The GUIDES Context Domain was linked to the largest number of belief statements (19/53; 35.8%). Each TDF domain linked to one or more GUIDES factor, with 6 TDF domains linking to more than 1 factor and 8 TDF domains linking to more than 1 GUIDES domain.

**Conclusions:**

The TDF provides unique insights into barriers and enablers to CDSS uptake, which can then be mapped to GUIDES domains and factors to identify required changes to CDSS context, content, and system. This can be followed by conventional mapping of TDF domains to behaviour change techniques to optimize CDSS implementation. This novel step-wise approach combines two established frameworks to optimize CDSS interventions, and requires prospective validation.

**Supplementary Information:**

The online version contains supplementary material available at 10.1186/s12911-023-02273-6.

## Background

Despite high-quality clinical practice guidelines for managing asthma [1, 2] knowledge-to-practice care gaps remain large, and asthma control remains suboptimal in up to 90% of patients [3]. Computerized clinical decision support systems (CDSSs) can address care gaps by leveraging evidence-based software algorithms to assist healthcare providers with clinical decision making [4]. However, poor uptake has limited the usefulness of these systems in real-world settings, and particularly in primary care [5, 6].

The Electronic Asthma Management System (eAMS) is an asthma management CDSS for primary care that was developed through an integrated knowledge translation approach, leveraging iterative end-user feedback and applying best evidence for effective communication of evidence-based guidance [7, 8]. In a clinical trial, the eAMS significantly improved all 3 key targeted asthma care practices in those settings [asthma control assessment according to guideline criteria; controller/reliever medication prescription ratio; and delivery of asthma action plans (AAPs)] [9]. These quality improvements were achieved despite suboptimal uptake, with the CDSS being accessed in 205 of 1033 (19.8%) possible instances and for 168 of 490 (34.3%) eligible patients over the 1-year study [7]. At the same time, an “on treatment” analysis demonstrated much larger improvements in those care practices in the subset of clinical interactions in which the system was actually used [9], suggesting that greater uptake would drive greater outcome improvements.

Recognizing that uptake is the first and necessary step for the success of any CDSS [10], and that changes in any targeted behaviours would likely correlate with initial uptake, we sought to apply the formal, theory-based Theoretical Domains Framework (TDF) approach to identify the determinants (barriers/enablers) of uptake of this eAMS CDSS. Next, we sought to match those determinants to elements in an evidence-based CDSS design and implementation framework - the Guideline Implementation with Decision Support (GUIDES) Checklist - to determine both the nature and extent of alignment between these frameworks, and the potential of this sequential approach to match identified determinants to system design “solutions” in an effort to improve system uptake.

## Methods

### Study design and setting

This was a descriptive qualitative study, complying with *Standards for reporting qualitative research* [11], conducted with primary care physicians recruited from the Greater Toronto Area.

### Sampling

We invited all 77 primary care physicians practicing across 5 sites as part of a large academic family health team in Toronto, and complimented this with purposive direct invitations sent to 8 physicians recommended by a study investigator (SG) based on existing clinical relationships. A brief informational email was sent to the family health team by a family health team administrative assistant on behalf of our research team, and to the eight recommended primary care physicians by the study research coordinator. The physicians were invited to contact the study research coordinator if they were interested in participating in the study. The research coordinator followed up by email with those physicians who expressed an interest in participating in the study. The only inclusion criterion was current use of an electronic medical record (EMR) system. As above, purposive sampling was used to ensure a balance of early (1st 5 years in practice) and later-career (> 5 years in practice), and academic and community-based clinicians. Each participant received a $50 gift card for participation. A minimum of 10 interviews are recommended for TDF studies, followed by initial analysis, and up to an additional three interviews or until data saturation is achieved [12].

### Theoretical approach

We applied a two-phased approach to conduct the study. In Phase 1, we used transcript analysis to derive belief statements in accordance with the TDF, characterizing the relevant domain for each statement and whether it represented a barrier or an enabler. The TDF is an integrative framework comprised of 14 theoretical domains derived from validated health and social psychology theories and constructs that may drive and explain health-related behaviour change from a psychological perspective [13, 14]. The interview guide was designed to explore which domains in the TDF were relevant for the targeted behaviour (usage of the CDSS by primary care physicians, in the primary care clinic, during a scheduled patient appointment) and how each of those domains influenced this behaviour [12, 15]. Supplementary file 1 describes the 14 TDF domains [13] and associated interview questions.

In Phase 2, we mapped belief statements and their corresponding TDF domains to relevant GUIDES domains and factors. The GUIDES is a framework of factors that contribute to successful CDSS interventions, developed through comprehensive CDSS literature review, expert input, and user consultation [16]. Sixteen factors that could affect the success of CDSSs are classified within four domains: context, content, system, and implementation. Given that the TDF identifies *determinants* of system use, and the GUIDES presents a framework for *optimal* CDSS design and implementation, we hypothesized that barriers and enablers to CDSS use from the TDF analysis would align with factors in GUIDES domains, with the latter presenting “solutions” (corresponding modifications) to leverage enablers and overcome barriers. Using belief statements as a bridge between corresponding TDF barriers and enablers and relevant domains and factors from the GUIDES checklist enabled us to determine to what extent and how elements within these two frameworks were aligned. The combined use of the TDF and GUIDES domains represents a novel marriage of two validated frameworks to derive specific insights on how to optimize CDSSs for better user uptake.

### Interviews

The semi-structured interview guide was initially developed by lead investigators (JY and SG), then reviewed by the interview team (JY, AK, SS) and revised for readability and relevance to the TDF domains. The interview procedure was pilot tested with members of the research team and adapted to ensure an approximately 45-minute duration. Approval was obtained from the Research Ethics Boards at Toronto Metropolitan University and St. Michael’s Hospital, Unity Health Toronto. Informed consent was obtained from all subjects. All methods were performed in accordance with the declaration of Helsinki.

After participants completed a demographic questionnaire, the interviewer explained and demonstrated eAMS functions, role, and workflow. This started with mock completion of the 5–10-minute electronic questionnaire which patients complete on a smartphone, tablet, or computer in advance of their clinical visit. Data from the questionnaire were then processed by the CDSS, which instantaneously presented clinicians with an EMR-integrated clickable prompt. Participants were asked to click on this prompt in a laptop/desktop computer, opening a CDSS window which presented a maximum of 5 screens (typically completed in 3–5 min in real-world use) (see Supplementary file 2 for further details). After system use, the trained interviewer proceeded with questions exploring each TDF domain (Supplementary file 1). Interviews were audio-recorded, de-identified and transcribed verbatim. NVivo 12 qualitative software (QRS International) was used to organize and code data.

### Analysis

Phase 1: We summarized participant characteristics descriptively and analyzed interviews using content analysis [17]. Two coders (JY and SNS) independently analyzed interview transcripts and each quote was coded deductively into the 14 TDF domains using a coding scheme developed by the coders. Each quote was then aligned with a belief statement representing a theme that was inductively generated from the data by the coders, and represented a core belief (i.e., barrier or enabler) [12]. The coders met on a regular basis to discuss and resolve discrepencies in coding. All belief statements corresponding to a TDF domain were considered for relevance, including conflicting or opposing beliefs within a domain, and any beliefs thought by the research team to influence CDSS uptake [12]. Verbatim quotes were used to represent the belief statements within each domain. Two reviewers (JY, SG) then confirmed the appropriate TDF domain for each belief statement.

Phase 2: The same two reviewers (JY, SG) independently mapped each belief statement to a corresponding GUIDES domain and factor. We calculated: the proportion of TDF belief statements that were linked to each of the 4 GUIDES domains; the proportion of TDF domains that linked to GUIDES factors (and vice-versa) and GUIDES domains, and which domains and/or factors remained unmapped. All discrepancies in coding and mapping were resolved through discussions between coders.

## Results

**Fig. 1:**
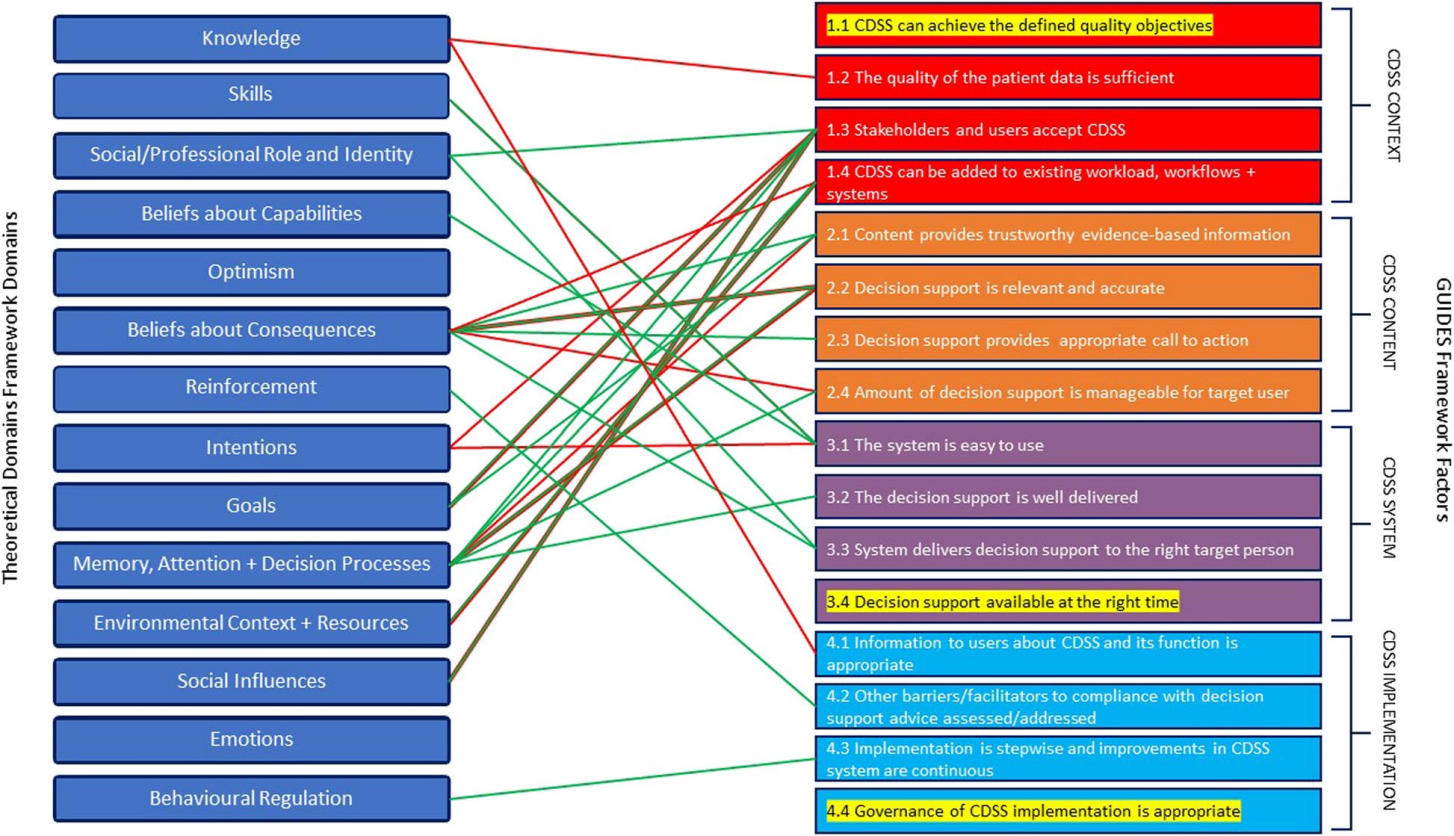
Mapping of Theoretical Domains Framework domains to GUIDES framework factors, on the basis of elicited belief statements relating to usage of the Electronic Asthma Management System (eAMS) CDSS. GUIDES factors are grouped by colour, according to the 4 GUIDES domains (CDSS Context, Content, System, and Implementation). When one or more belief statement(s) linking a TDF domain to a GUIDES factor was characterized as an enabler to system use, the link was depicted as a green line, whereas when one or more belief statement(s) was characterized as a barrier to system use, the link was depicted as a red line. GUIDES factors that were not linked to any TDF domains are depicted in yellow highlight. TDF belief statements that did not link to a specific GUIDES factor are not depicted. CDSS = clinical decision support systems; GUIDES = Guideline Implementation with Decision Support; TDF = Theoretical Domains Framework

 Of the total 85 primary care physicians invited to participate in the study, 10 initially consented, and data saturation was achieved after those 10 interviews. Participant characteristics are summarized in Table 1. Twelve of the 14 TDF domains were considered relevant, with most belief statements representing enablers to CDSS uptake. Two of the TDF domains (Optimism and Emotions) were not considered relevant. A summary of belief statements, assigned TDF domains, corresponding participant quotes and associated GUIDES checklist domains and factors is provided in Table 2. Of the 53 belief statements, 18 (34.0%) were barriers and 35 (66.0%) enablers Forty-one statements (77.4%) linked to at least one GUIDES factor. The remaining 12 (22.6%) statements were categorized as pertaining to one or more of the four broad GUIDES domains. Overall, 19/53 (35.8%) of belief statements linked to the GUIDES Context Domain, 16/53 (30.2%) to the Content Domain, 14/53 (26.4%) to the System Domain, and 5/53 (9.4%) to the Implementation Domain (one belief statement linked to two GUIDES Domains). The GUIDES Context Domain linked to the largest number of TDF barriers (9/18, 50.0%) and the Systems Domain to the largest number of TDF enablers (12/35, 34.3%). Figure 1 maps the relationships between TDF domains and GUIDES factors. All TDF domains linked to one or more GUIDES factor, with 6 TDF domains linking to more than 1 GUIDES factor and 8 TDF domains linking to more than 1 GUIDES domain (with some TDF domains linking with up to 3 GUIDES domains). The TDF domains linked to the most GUIDES factors (6 TDF domains) were *Memory, Attention and Decision Processes and Beliefs about Consequences*. Almost half of the GUIDES factors (7/16, 43.8%) linked to more than one TDF domain, with CDSS Context: 1.3 Stakeholders and users accept CDS linking to the most TDF domains (5 domains). There were 3 GUIDES factors that were not raised in any belief statement: CDS Context: 1.1 CDS can achieve the defined quality objectives; CDS System: 3.4 The decision support is available at the right time; and CDS Implementation: 4.4 Governance of the CDS implementation is appropriate. This is likely a reflection of our specific interview/study process (for factors 1.1 and 4.4), and of our CDSS design (for factor 3.4). Factor 1.1 CDS may not have been raised because the evidence base for the content and effectiveness of the eAMS CDSS was clearly presented to participants during the interview preamble (Supplementary file 1). Factor 4.4 may similarly not have been a concern because most interviewees were familiar with research team leadership and recognized their role and position within the academic institution. Finally, factor 3.4 was likely irrelevant because of our CDSS integration within the EMR, at the point-of-care and during the clinical interaction.

**Table 1:** Participant characteristics of (*n* = 10)

Variable	n (%) or Mean ± SD
Age (years)	
25–30	1 (10.0)
31–40	6 (60.0)
41–50	2 (20.0)
51–60	1 (10.0)
**Sex**	
Female	6 (60.0)
**Early career (1st 5 years in practice)**	4 (40.0)
**Later career (> 5 years in practice)**	6 (60.0)
**Work in an academic centre**	5 (50.0)
**Work in a community setting**	5 (50.0)
**% of time spent on clinical activities**	76.2 **±** 28.7
**# of asthma patients seen each month**	6.3 **±** 3.4
**Number of years using an EMR**	8.8 **±** 3.7
**Currently use a CDSS in EMR**	5 (50.0)

*EMR *Electronic medical record, *CDSS* Computerized decision support system

**Table 2:**
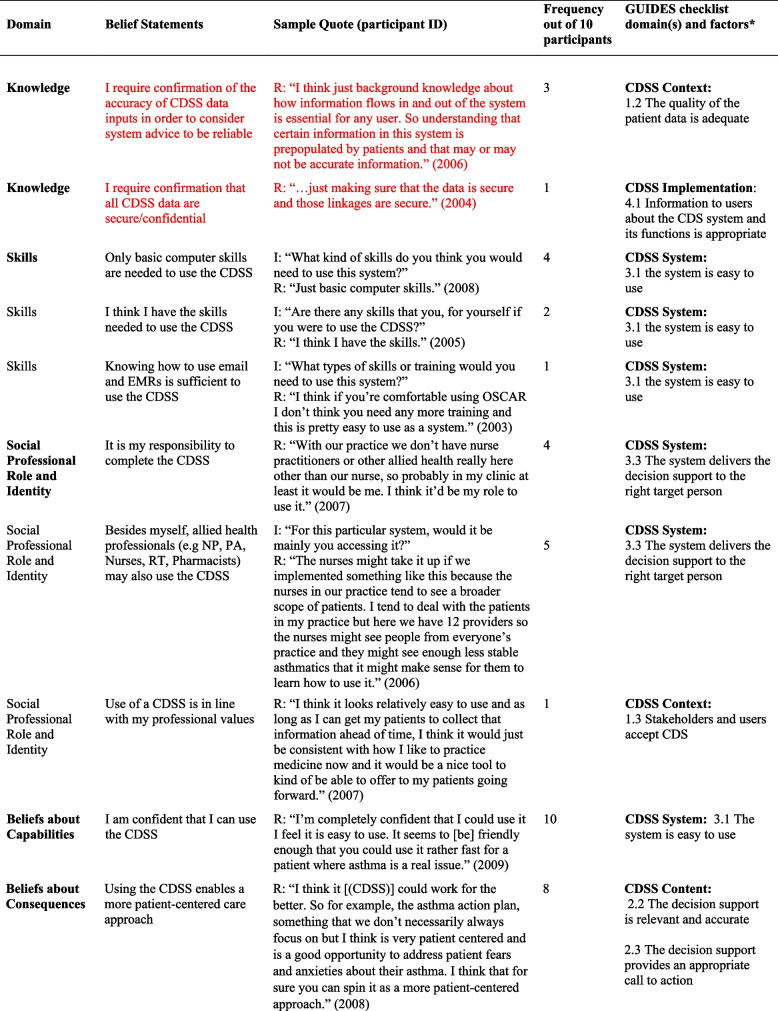

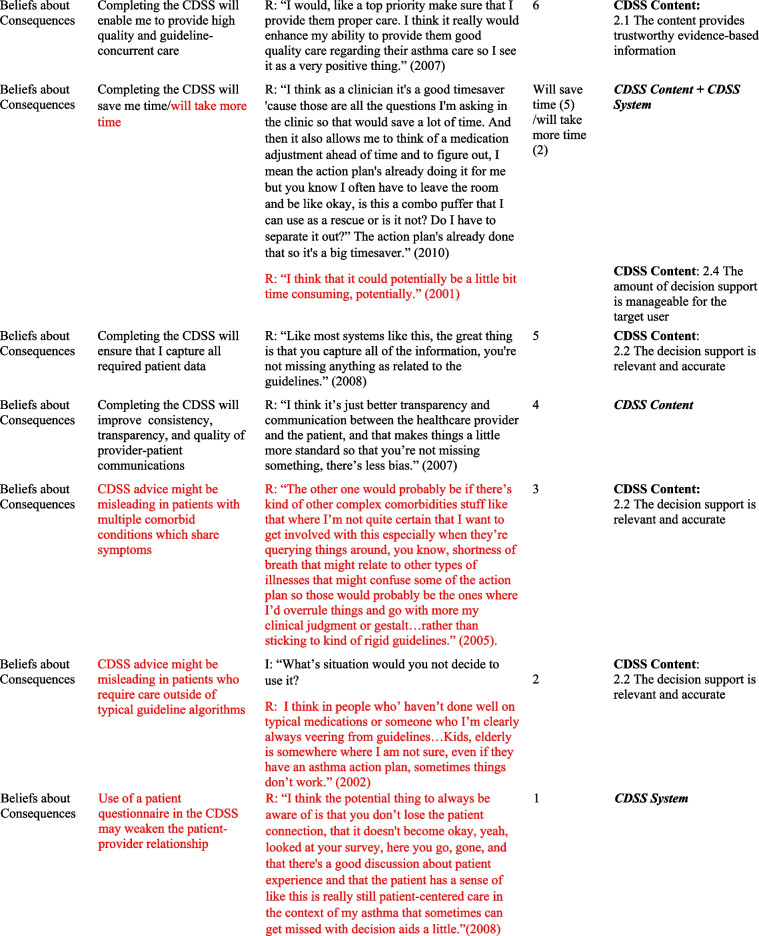

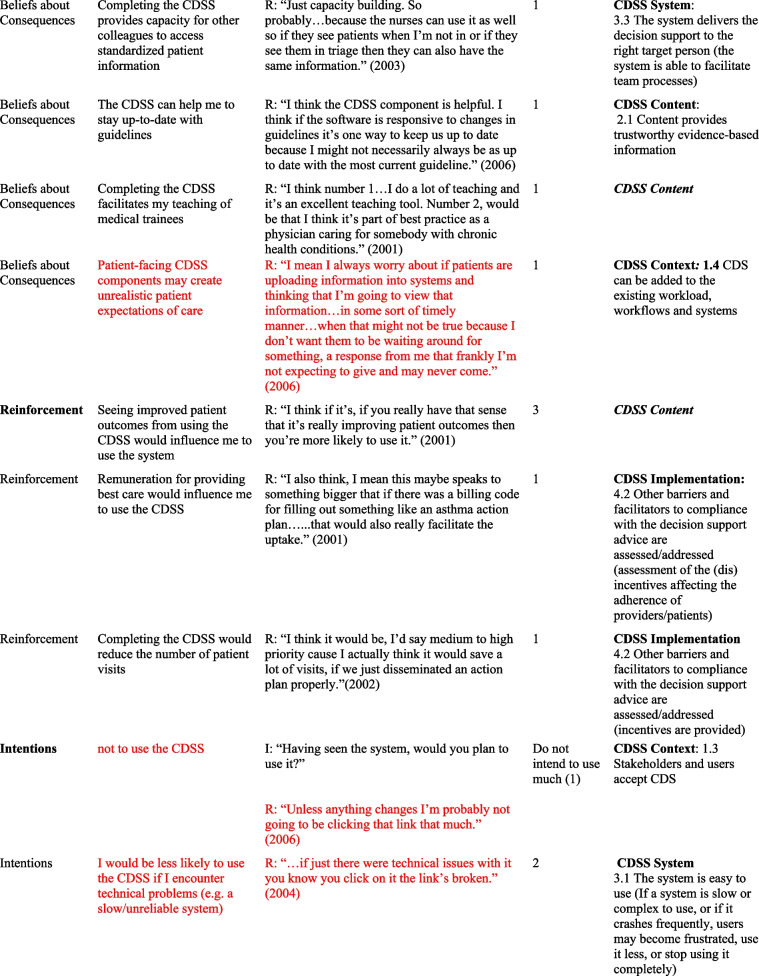

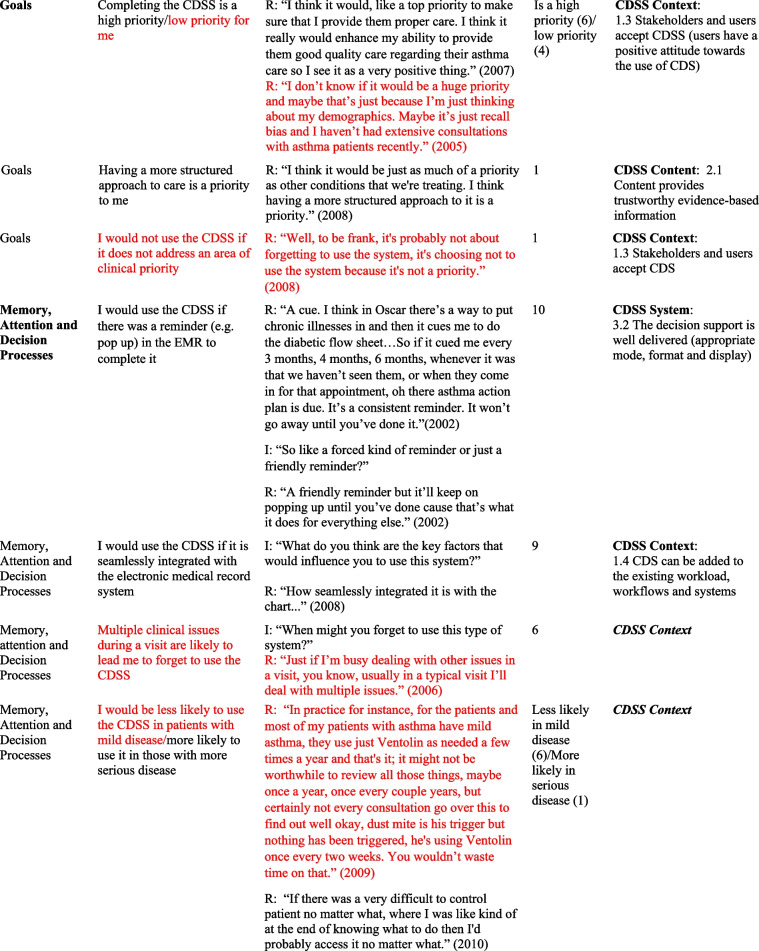

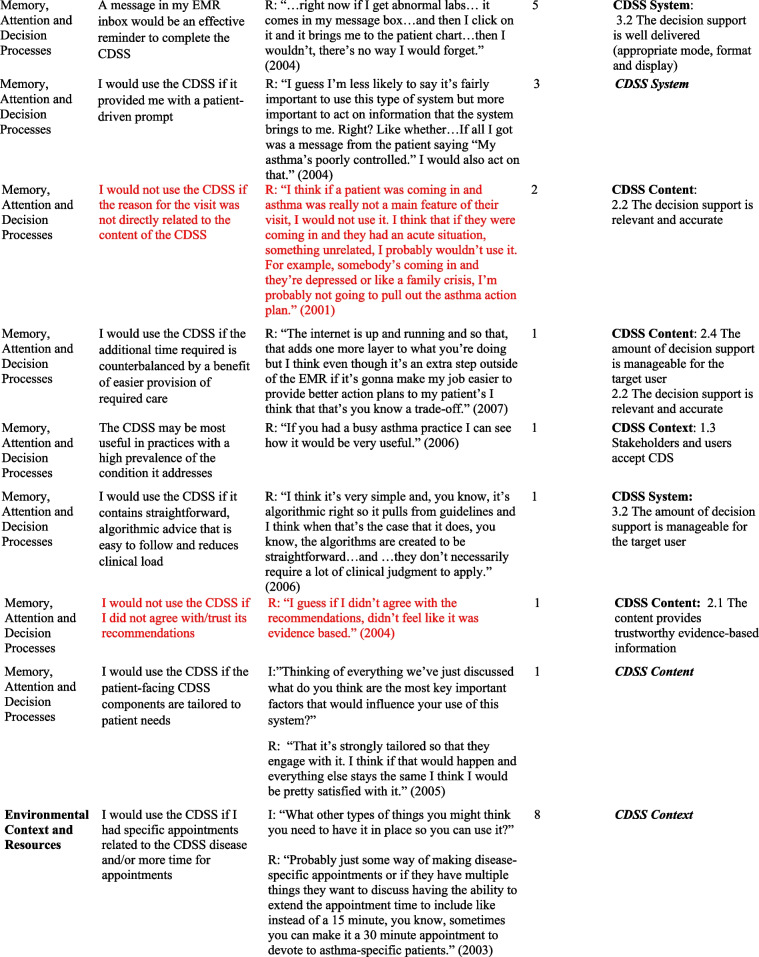

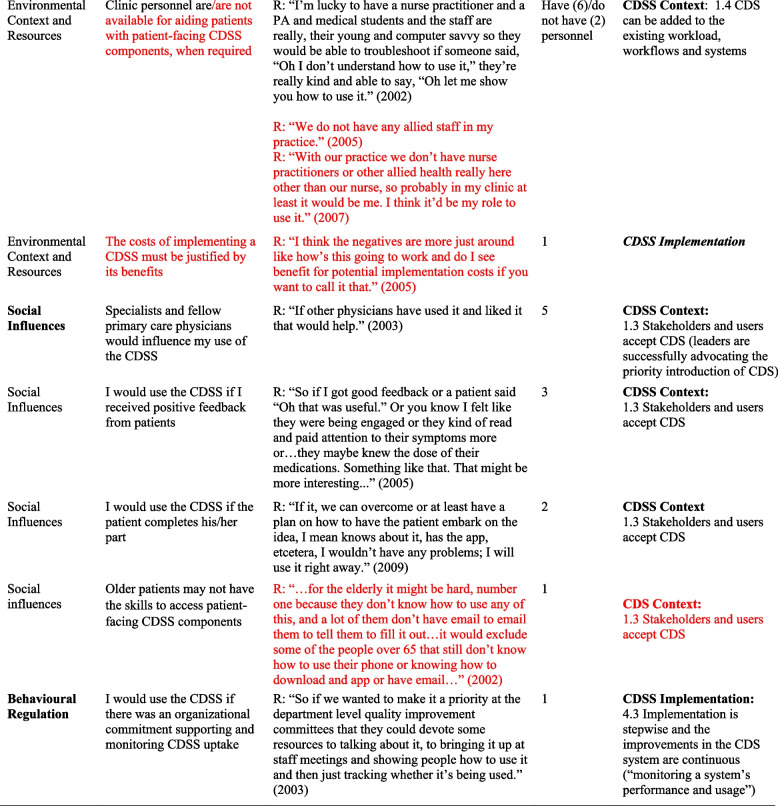
Relevant TDF domains, belief statements, sample quotes and associated GUIDES domains and factors

*I* Interviewer, *R *Respondent, *TDF *Theoretical Domains Framework* Barriers in red font

*In some cases, in addition to factors, factor subdefinitions are provided in brackets for clarification. Also, some belief statements did not map to specific factors; in those cases, we provide only the domain that we deemed most appropriate (***italicized*** for distinction)

Our analysis yielded several insights about the barriers and enablers to use of the eAMS and corresponding required intervention adaptations. A large number of reported determinants of system use (6/18 identified barriers and 8/35 enablers) pertained to the patient-facing pre-visit questionnaire in the eAMS. Barriers included concerns about information reliability, with providers perceiving that older patients might lack the skills to complete an electronic questionnaire (*Social influences*) and providers requiring advance *Knowledge* that patient inputs were accurate. Corresponding GUIDES factors call for ensuring that the quality of the patient data is adequate (CDSS Context). The questionnaire also raised concerns about the impact on provider-patient interactions, with *Beliefs about Consequences* including weakening of the patient-provider relationship and creating unrealistic patient expectations of care. The former did not align with a specific GUIDES factor, and the latter corresponded with ensuring that system requirements can be added to the existing workload (CDSS Context). In this case, this might be achieved by setting patient expectations through messaging within the questionnaire. Limited *Environmental Context and Resources* (i.e. staff) for aiding patients with patient-facing components, and security of the transmitted patient data (i.e. providers requiring *Knowledge* of adequate data security protocols) were also limitations. As per GUIDES factors, the former requires optimization of CDSS Context by ensuring that the CDSS can be added to the existing workflows, and the latter improving on CDSS Implementation by providing upfront information to users about system security.

However, at the same time, use of a patient-facing questionnaire presented several enablers to CDSS uptake. Providers recognized that advance data collection would save them time (*Beliefs about Consequences*) – a concept that did not directly match to any GUIDES factors but could be emphasized to providers to drive uptake. Also, what was an *Environmental Context and Resources* barrier for some (staff availability, above) was an enabler for others, where the CDSS Context featured sufficient clinic staff for any required patient assistance and where this was recognized as a worthwhile use of resources. Users also saw this as an opportunity to offer more patient-centered care (*Beliefs about Consequences*), fulfilling the GUIDES requirements for CDSS Content to be relevant and to provide an appropriate call to action. Similarly, users would use the system (*Memory, Attention and Decision Processes*) if the patient-facing CDSS components were tailored to patient needs – another concept that did not directly align with a GUIDES factor but could be used to promote system value. Some also indicated a perceived benefit that this approach would capture all required patient data (*Beliefs about Consequences*), aligning with the GUIDES factor describing the importance of relevant and accurate CDSS Content. It was also noteworthy that a patient-facing component was seen as a potential driver of provider uptake, through a patient-driven prompt (*Memory, Attention and Decision Processes*) – a concept not explored in GUIDES factors. A related finding was that providers would be more likely to use the system if patients had completed their part, and particularly if they had received positive feedback from patients (*Social Influences*) – both of which related to the GUIDES CDSS Context factor calling for *all *stakeholders and users to accept the system.

We also noted several determinants of system use that were related to the specific patient population being addressed. Providers indicated concerns about the accuracy of CDSS guidance in patients with comorbid conditions and/or those requiring care outside of typical guideline algorithms. Both of these adverse *Beliefs about Consequences* could be addressed through GUIDES CDSS Content factors relating to the relevance and accuracy of the content (e.g. by including guidance for special populations or by clearly identifying and acknowledging populations in which guidance should not be used). Another reported barrier was the perception that the system would not be needed in patients with mild disease, with the corresponding enabler being that providers would be more likely to use it in those with more serious disease (*Memory, Attention and Decision Processes* ***)***. These are CDSS Context-related issues, suggesting that implementers may either choose to promote use only in patients with more severe disease, or focus educational efforts on relating the health benefits of bridging targeted practice gaps even in patients with mild disease.

Along similar lines, several determinants surrounded the nature of the specific patient visit and the broader nature of the provider’s practice. Providers indicated that multiple clinical issues during a visit might lead them to forget to use the CDSS for a specific disease (*Memory, attention and Decision Processes*) (an issue that is broadly relevant to the CDSS Context, without a specific pertinent GUIDES factor), and they would not use it if the reason for the visit was not directly related to the content of the CDSS (*Memory, Attention and Decision Processes*) (addressed under GUIDES CDSS Content – ensuring that the decision support is relevant). On the other hand, they indicated a high likelihood of system use if the patient had a visit dedicated to the CDSS disease (*Environmental Context and Resources*) (broadly relevant to the CDSS Context, without a specific pertinent GUIDES factor). Practice-related barriers included a low overall priority to use the system due to a low prevalence of the targeted condition in the provider’s practice (*Goals*), and the condition not representing an area of clinical priority (*Goals*), both relating to GUIDES factors describing the importance of positive user attitudes towards the CDSS and user acceptance of the CDSS (CDSS Context). This was accompanied by a perception that such a system would be most useful in practices with a high prevalence of the condition it addresses (*Memory, Attention and Decision Processes*), again relating to the GUIDES factor emphasizing the importance of stakeholders’ and users’ acceptance of the decision support (CDSS Context).

Finally, many determinants of use surrounded the availability of sufficient time to interact with the CDSS. A belief that use would require additional visit time  was a barrier, addressed by the GUIDES CDSS Content requirement for developers to ensure that the amount of decision support is manageable for the target user. Interestingly, some users perceived time *saved* through system use to be an enabler (*Beliefs about Consequences*) (a concept pertaining to CDSS Content and CDSS System but not addressed by a specific GUIDES factor). A perception that time required for system use was adequately “counterbalanced” by the benefit of easier provision of required care was also seen as an enabler (*Memory, Attention and Decision Processes*), relating to GUIDES factors calling for relevance of the decision support and a manageable amount of decision support for the target user (CDSS content concepts). User perceptions of their *Social Professional Role and Identity* were also an enabler, including a belief that CDSS use aligned with their professional values (addressing a GUIDES CDSS Context factor surrounding stakeholders’ and users’ acceptance of the CDSS), and that when qualified allied health staff were not available to do so, it was physicians’ responsibility to complete the CDSS (in line with a CDSS System requirement to deliver the decision support to the right target person).

## Discussion

To our knowledge, this is the first study to apply the TDF to assess barriers and enablers to using a CDSS, and to attempt to identify strategies for intervention improvement by mapping those determinants of uptake to a framework for CDSS intervention design (the GUIDES Framework).

Unique insights were provided by assessing the identified determinants of CDSS system use and their linkage to GUIDES constructs. Our finding that the pertinence of the reason for the clinical visit to the content of the CDSS was an important determinant of system use aligns with a multivariable model in which the strongest predictor of both accessing the eAMS and completing all eAMS steps was a respiratory presenting complaint [7]. Prioritizing decision support at the “moment of need” aligns with previous studies of determinants of CDSSs success [18, 19]. Although chronic disease management CDSSs are designed to enable maintenance care that *prevents* acute events, our data suggest that system usage will be constrained by acute patient priorities. In our prior study, a third of asthma patients presented with respiratory complaints over one year [7]. For diseases such as this, it might thus be reasonable to follow the GUIDES CDSS Content recommendation to ensure that the decision support is highly relevant - leveraging this enabler by triggering decision support only during visits for corresponding acute complaints. However, CDSSs designed to address screening or management of asymptomatic risk factors (e.g. hypertension) will require alternative strategies. Given guidelines calling for periodic preventive health visits to replace traditional annual physical examinations [20], tailored preventative visits could address the identified barriers while also leveraging an identified enabler - a dedicated visit for CDSS use (thereby optimizing the GUIDES CDSS Context). This also highlights how CDSS Content and Context solutions related to the same barriers and enablers will vary as a function of the disease being addressed.

Our findings that numerous determinants of system use surrounded user perceptions of value-for-time align with prior reports that time constraints remain a barrier to uptake even when users are aware that CDSSs improve care [21, 22]. In the eAMS trial, in 34% if instances of CDSS usage, the system was accessed *after* the patient appointment [7], indicating that even providers who valued the tool often could not utilize it while facing clinical demands. Driving system use will thus require increasing the perceived value, decreasing the required time, or both. This aligns with the GUIDES requirement to ensure that content is both manageable in its quantity and relevant in its quality. Some users considered system use an *additional* time burden for completion of low priority “add-on” tasks, while others were enticed by the time *saved* relative to the conventional workflows they employed to complete perceived “essential” tasks. Accordingly, user perceptions of task importance play a key role in whether time acts as a barrier or enabler to CDSS use. Although the GUIDES framework emphasizes minimizing *additional time* required for decision support, it does not contain advice to frame system use as time-*saving*. This enabler could be leveraged by attempting to change the perceived importance of a CDSS-enabled task by conveying patient-relevant health impacts expected to result from it and/or through direct audit and feedback reporting on patient improvements. Indeed, providers reported that the reinforcement received from seeing improved patient outcomes from CDSS use would act as an enabler to use, which was a concept broadly relevant to CDSS Content but not specifically addressed in the GUIDES. One might also consider designing a system that can be adapted to different users’ task prioritizations, allowing for both limited and extensive use cases and preference-based rather than linear task completion pathways. Furthermore, given the growing prevalence of multi-chronic disease co-morbidities [23], the “one issue per visit” axiom [24] may prove beneficial, whereby the CDSS might employ software algorithms to “select” which chronic disease should be prioritized in a particular visit, or provide the clinician with that choice (a CDSS System feature).

Incentives might also be used to directly alter the value component in the value-for-time equation. Participants identified remuneration for CDSS use as an enabler, aligning with “incentives” in the GUIDES CDSS Implementation domain. Similarly, reducing the number of patient visits (equating to a financial incentive in capitation model-remunerated participants) was an enabler addressing the same GUIDES factor [25]. Finally, provision of continuing professional development credits could also carry an implied monetary value, encouraging system use.

The largest number of identified TDF barriers linked to the GUIDES Context domain and largest number of TDF enablers to the Systems domain. This suggests that to optimize uptake, the Context of intervention use will require the most adjustment, whereas System features offer the most leveraging opportunities. Six TDF domains linked to more than one GUIDES *factor* (up to 6), and 8 linked to more than 1 GUIDES *domain* (up to 3). This reinforces the complexity of successful CDSS intervention design, whereby addressing or leveraging a single *type* of behavioural determinant will not only often require changes to numerous conceptually similar intervention features (i.e. within a GUIDES domain), but also to disparate intervention features, across domains. For example, our findings regarding users’ *Beliefs About Consequences* of CDSS use indicate the need for: a strategy to address concerns about the time required to complete the CDSS (ensuring a manageable amount of decision support - a CDSS Content factor); efforts to mitigate the potential for a patient-facing questionnaire to create unrealistic patient expectations of care (such that providers believe the system can be added to the existing workload – a CDSS Context factor*);* and reinforcing that CDSS use will allow for one’s colleagues to access standardized patient information (whereby the system is able to facilitate team processes- a CDSS System factor).

Almost half of the GUIDES factors also linked to more than one TDF domain, suggesting that a single change to the CDSS intervention can impact different behavioural determinants of system use. For example, CDSS content factor 2.2, whereby decision-support should be relevant and accurate, was linked to TDF domains *Beliefs About Consequences* (e.g. belief statement “Completing the CDSS will ensure that I capture all required patient data”), and *Memory, Attention and Decision Processes (*e.g. belief statement “I would not use the CDSS if the reason for the visit was not directly related to the content of the CDSS”). Accordingly, changes to the system that might impact perceived relevance and accuracy of advice content could impact behavioural determinants surrounding at least 2 theoretical domains. The GUIDES factor associated with the most TDF domains (5 domains) was “Stakeholders and users accept CDS” (CDSS Context), reflecting the multiplicity and diversity of behavioural factors that might act both as barriers and enablers to *acceptance* of this complex intervention.

Finally, the same concept could act as both a barrier and an enabler, depending on the user. These “opposing” belief statements lined up almost exclusively with CDSS Context-related factors and were mostly dependent on the user’s practice environment. For example, the pre-visit questionnaire was a barrier to system use in settings with insufficient clinic staff to support patients, yet an enabler in settings where staffing was sufficient. This finding emphasizes both the importance of understanding the context in which the intervention is being launched and the potential value of tailored, context-specific intervention branches. We also noted that whether the concept underpinning the belief statement acted as a barrier or enabler could sometimes lead to a distinct corresponding GUIDES factor. This suggests that intervention changes required to overcome a barrier in some situations will be different than those that could be used to leverage the same concept as an enabler in others.

Of the 12 belief statements that could not be linked to a specific GUIDES factor, three related to how the context of clinical visits could influence provider CDSS use (the number of clinical issues addressed during a single visit, a patient’s disease severity, and the time available to address the CDSS-targeted condition). Though GUIDES generally addresses the question of workload feasibility, these beliefs statements may highlight a need to include more nuanced contextual factors related to individual patient/visit complexity. Insufficient consideration of such contextual factors in CDSS design and implementation was believed to partly explain the small effect sizes achieved by CDSS interventions in a recent meta-analysis [26]. Another belief statement not linked to a GUIDES factor was the positive influence of seeing improved patient outcomes from CDSS use. Performance feedback on CDSS use and care quality was identified as a factor of potential interest in a prior Delphi study [4], and feedback on *patient* outcomes may prove even more meaningful [27]. Finally, although the GUIDES calls for ensuring that the quality of patient data inputs is adequate, it does not address identified belief statements pertaining to patient-facing data collection interfaces as part of the CDSS, including: how patient questionnaires might enable time savings in clinical data collection, their effects on patient-provider relationships; how well they are tailored to patient needs; and how they might enable patient prompts for providers to use the CDSS. These factors are equally absent in other CDSS evaluation frameworks, despite the growing use of pre-visit electronic patient questionnaires, which have been shown to be a reliable, acceptable and usable in real-world settings [28], and present opportunities for patient-mediated knowledge translation [29, 30]. As CDSSs continue to evolve to include more patient-reported data, this should be a consideration in CDSS intervention development [31]. More broadly, identification of several potentially important belief statements which underpin system use through the TDF analysis, which are absent in the GUIDES framework, demonstrate the value of our unique approach, and the complimentary nature of these approaches (i.e. these factors would have been missed in a GUIDES analysis alone).

**Fig. 2:**
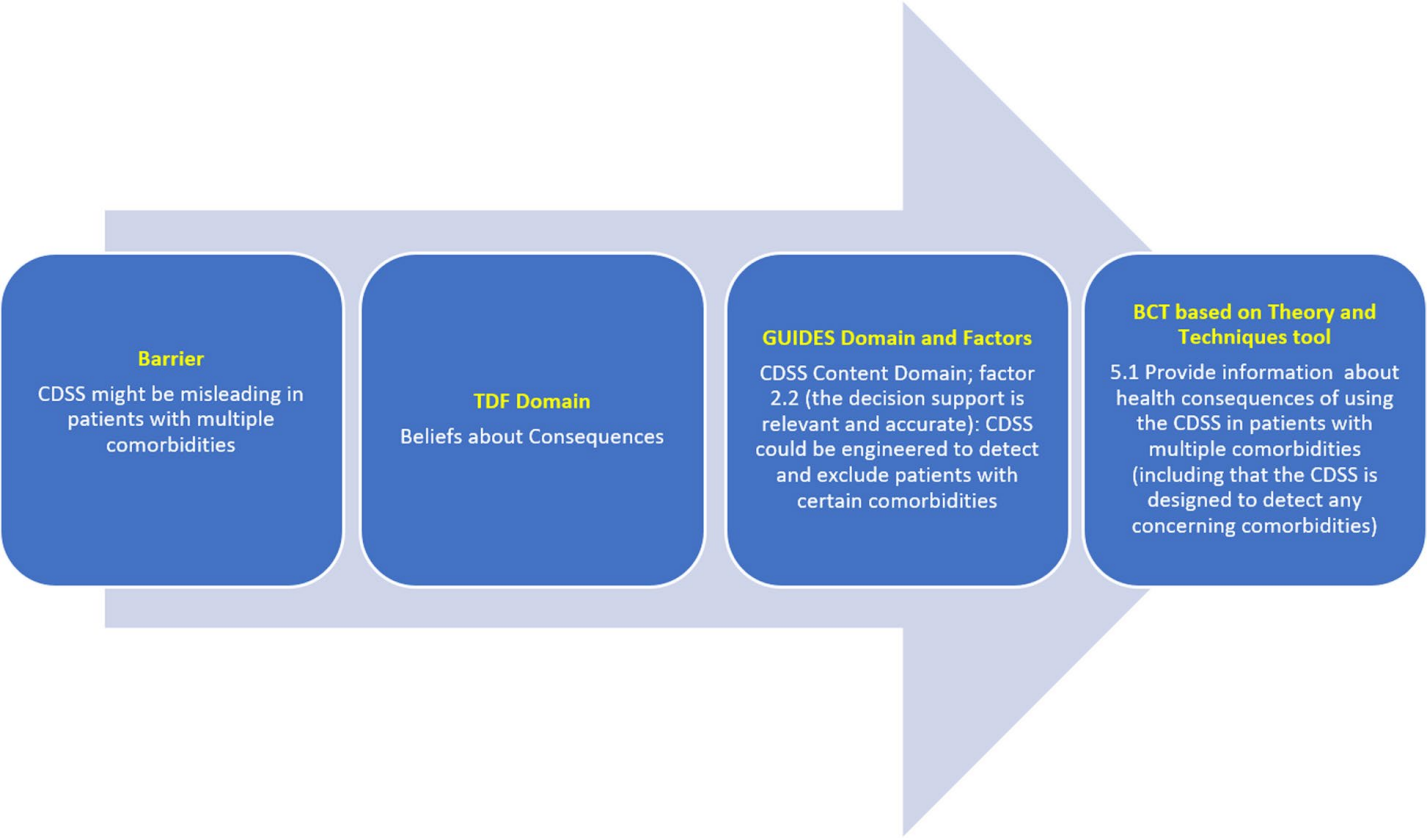
Proposed phased approach to optimizing a CDSS intervention *CDSS* clinical decision support systems, *BCT *Behaviour change technique, *TDF *Theoretical Domains Framework

A theory-based approach to developing successful behavioural interventions requires identifying domains in the TDF influencing a particular behaviour and matching these to corresponding behaviour change techniques (BCTs) [32, 33]. However, we believe that CDSS usage is conceptually different than “typical” provider behaviors of interest such as testing or prescribing, as the desired action itself is use of a complex software system, whereby uptake may be as strongly influenced by the context, content, and design of the system *itself*, as how it is implemented. Indeed, the majority of belief statements in our study were linked to Context, Content and System GUIDES domains, whereas only 5/53 (9.4%) were linked to the Implementation Domain. Previously, Camacho and colleagues [34] proposed a conceptual framework that could be used to improve of CDSS implementation by connecting factors relating to behaviour change to factors relating to CDSS technology acceptance. We propose a phased approach to optimizing CDSS uptake through existing validated tools. First, the TDF process could be used to identify barriers and enablers to uptake. Next, as an intermediate step between the TDF process and matching to BCTs required for implementation, the GUIDES checklist could be used to identify required changes to the CDSS technology and opportunities to optimize the intervention context (by addressing TDF belief statements mapping to the GUIDES domains). Finally, all TDF domain belief statements could be matched/linked to BCTs to optimize implementation, through a tool such as the Theory and Techniques Tool (Fig. 2) [35]. The Theory and Techniques Tool is an online tool that links 74 BCTs from a taxonomy to 26 mechanisms of action (including the 14 domains in the TDF). The tool was based on a comprehensive review of the literature and expert consensus and can be used to develop evidence-based interventions [36–38]. The strength of evidence for each BCT’s effect on each TDF domain is colour coded within the tool (https://theoryandtechniquetool.humanbehaviourchange.org/tool) [36].

Although we have not focussed on BCT matching in this report because it is well-described elsewhere [35], the following is an example of how these complimentary frameworks could both be applied. The TDF *Beliefs about Consequences* domain and associated barrier that CDSS advice might be misleading in patients with multiple comorbidities, aligns with the GUIDES CDSS Content Domain and factor 2.2 (“the decision support is relevant and accurate”). In response, the CDSS could be engineered to detect and exclude patients with certain comorbidities. At the same time, based on the Theory and Techniques Tool, a BCT with evidence of effect for the *Beliefs about Consequences* TDF domain was linked to specific information (written, verbal, visual) about health consequences (in this case) of following the CDSS advice in patients with multiple comorbidities (for example, informing users that these use cases are automatically detected and accounted for by the system) [36–38].

Our study has several strengths and limitations. Our approach enabled an exploration of the relationships between elements in the TDF and GUIDES frameworks and provides a successful test case for our novel proposed strategy for theory-based CDSS intervention optimization. Data were collected from primary care providers, and determinants of use may vary by healthcare user type. Similarly, determinants may vary across settings such as specialty and in-patient care. We also note that several determinants of use reported by providers were based on their perceptions of patient experiences with the system. To validate whether these perceptions were accurate, we cross referenced corresponding belief statements with those derived directly from a prior TDF analysis of patient-reported determinants of use of the eAMS [39]. Although 6 of 9 provider perceptions were validated by patient report, the remaining three, all relating to provider perceptions of the impact of the patient-facing questionnaire, were not reported by patients (Supplementary file 3). Finally, we demonstrated our proposed model in a single CDSS addressing a specific disease (asthma), and it now requires testing with other systems and in other diseases.

## Conclusions

In summary, we applied the TDF framework to assess barriers and enablers to using a CDSS, then matched extracted belief statements to elements in the GUIDES framework, enabling a mapping of TDF domains to GUIDES factors. Matched GUIDES factors yielded multiple insights relating mostly to how the context of CDSS use and the CDSS itself (how the system functions and its content) could be optimized for user uptake. We believe that GUIDES domain matching is a useful intermediate step between the TDF process for identification of behavioural determinants and the subsequent matching of TDF domains with BCTs for optimizing CDSS implementation. This proposed approach now requires both qualitative validation determining user-perceived value of system and intervention changes produced, and quantitative validation measuring changes in user uptake.

### Supplementary Information

**Additional file 1.** Asthma Study Interview Guide for Physicians

**Additional file 2. ** eAMS Demonstration

**Additional file 3.** Statements Describing Physician Perceptions of Patient Experience

## Data Availability

The authors confirm that all relevant data are included in the paper and quotes from study participants are included in the supplementary table. Original transcripts from the current study are available from the corresponding author upon reasonable request and research ethics approval for release.
