# Supplementary material for: Improving computerized decision support system interventions: a qualitative study combining the theoretical domains framework with the GUIDES Checklist
Source: BMC Med Inform Decis Mak. 2023 Oct 18;23:226. doi: 10.1186/s12911-023-02273-6 (PMC10585867; doi:10.1186/s12911-023-02273-6)
Supplement: Supplementary file 1 — Additional file 1. Asthma Study Interview Guide for Physicians [file 12911_2023_2273_MOESM1_ESM.docx]

**Supplementary File 1: Asthma Study Interview Guide for Physicians**

Completion of a computerized clinical decision support system (CCDSS) for asthma management in primary care.

Thank you for agreeing to participate in a discussion about our Electronic Asthma Management System (eAMS). The system was developed to assist primary care clinicians in managing asthma in adults. Given that over 75% of Canadian primary care physicians now use electronic medical record systems (EMRs), we designed and integrated the system into the EMR, in this case the OSCAR EMR as a test case. Our system consists of three parts.

1. An electronic questionnaire which collects asthma parameters from patients in advance of their clinical visit. The questionnaire takes about 10 minutes to complete. It can be done at home on a smartphone, ipad, or PC, or in the clinic waiting room on the patient’s phone or an iPad provided by the receptionist. The questionnaire was developed and tested through serial focus groups with asthma patients. Patients get a message through the questionnaire app if they have already downloaded it to their phone or tablet, or through a text or email message 1 week before their appointment with a reminder to complete the questionnaire.
2. A point-of-care computerized clinical decision support system that receives and processes questionnaire data to produce decision support for the clinician, in accordance with the Canadian Asthma Guidelines. This includes an assessment of the patient’s current asthma control level, corresponding medication change recommendations, and a personalized self-management asthma action plan, all of which is integrated into the EMR in real-time.

The clinician interface was designed by a group of both academic and non-academic primary care physicians. After interacting with the decision support system, clinicians can access and print an asthma action plan from the EMR in order to deliver it to the patient, and a chart note detailing all actions in the decision support system. Any new required prescriptions are automatically written to the chart. Clinicians can then create any new required prescriptions and provide these to the patient.

1. The asthma action plan and medications are also sent to a patient-facing app called “breathe” which is integrated with the system and helps patients to manage their asthma through such features as air quality warnings, medication reminders, and logging of triggers and symptoms.

We pilot tested the system at 4 primary care sites in Ontario over 1 year and found significant improvements in asthma control assessment and delivery of asthma action plans (assessment improved from 5% of visits to 28% of visits, and action plan delivery from 0.2% of patients to 18% after the system was in place). However, clinicians did not consistently access the system when it was available to them.

Accordingly, the aim of our project is to evaluate clinician views about using this tool in their primary care setting. Our discussion should take approximately 30-45 minutes and will be audio-recorded to ensure that all key points are documented. You will be shown a demonstration about the system followed by an interview. Any identifying information (for example the names of other individuals) that you use in the course of our discussion will be removed from the interview transcripts. After the interview, we would like you to complete a brief demographic questionnaire that includes questions about your practice.

If you would like to end the interview before I have asked all of the questions or if you wish to withdraw at any time from the study, you are free to do so.

Understanding your views will help us to ensure that the system is user-friendly and will assist us to make adjustments to this system to promote its usage. There may be overlap and repetition of some of the questions; however, each question is worded to obtain specific information. It is also important to note that there are no right or wrong answers. Your views will assist us in improving the system and its uptake.

Do you have any questions before we start?

**Introduction to the System**

The physician portion of the system takes approximately 5 minutes to complete.

I will start by showing you a demonstration of how the system works

What are your overall views about the system?

What do you think works well?
What do you think does not work well?

We have specific questions for you on the use of the doctor portion of the system.

Targeted behaviour: Using the eAMS system

| **TDF** | **Interview Question** |
| --- | --- |
| Knowledge | Having seen the system, do you need more information about how to use it or why it should be used?  Do you feel that you would need to practice in order to use the system?  What would it take for you to use the system? |
| Skills | What types of skills training or guidance (if any) do you think you would need to use the system? |
| Social Professional Role and Identity | Whose role would it be to use the system? Do you feel that it is part of your role/job to use the system? Are there other health professionals who should use the system in addition to you or instead of you? |
| Beliefs about Capabilities | How easy/difficult do you think it would be to use the system? What parts of the system would be easy/challenging to use? How confident do you feel in your ability to use the system? What would make you feel more confident in your ability to use the system? |
| Beliefs about Consequences | What do you think are the benefits to using the system for yourself as a clinician, for your patients, for your professional colleagues, and for your practice? What are the good things and bad things that can happen from using this system? |
| Optimism | How likely do you think it is that using the system will lead to better asthma management? Do you think this system will work? Why/Why not? |
| Intentions | Having seen the system, would you plan/want to use it? |
| Memory Attention and Decision Making Processes | Could the system become integrated into your current workflow or would it be something you would need to stop and take time to think about each time you use it?  What would help you to integrate using system into your current workflow?  In what situations would you decide/ not decide to use the system? Why? What would you do instead of using the system, for those particular elements of care?    When might you forget to use the system? Why? What could help prevent this? |
| Environmental Context and Resources | Is there anything in your work environment/practice that might influence your use of the system?  Are there any competing tasks or time constraints that could influence your use of the system?  How could you address these issues?  What resources do you have in your practice to support the use of such a system? What other resources do you think you would need in order to use such a system? |
| Social Influences | Who could influence you to complete the system (e.g., colleagues, patients)? |
| Emotions | Tell me how you feel about using the system. Would you have any worries/concerns about using it? |
| Goals | How much of a priority would it be to use such a system in your practice compared to other priorities you may have? |
| Behavioural Regulation | What do you think would help/what strategies should be in place (e.g. monitoring) to ensure that you use the system? |
| Reinforcement | How would your experiences (good or bad) of using such a system influence whether or not you would use it again? Are there any incentives or might there be certain rewarding experiences that would increase your likelihood of using the system? |

Final question: Thinking of everything we have just discussed, what do you think are the most important factors that would influence your use of the system?
