# Supplementary material for: Improving computerized decision support system interventions: a qualitative study combining the theoretical domains framework with the GUIDES Checklist
Source: BMC Med Inform Decis Mak. 2023 Oct 18;23:226. doi: 10.1186/s12911-023-02273-6 (PMC10585867; doi:10.1186/s12911-023-02273-6)

**Supplementary File 2: eAMS Demonstration**

**Methods**

## Interviews

When participants clicked on the system prompt in the laptop/desktop computer, it triggered a CDSS window which presented users with a maximum of 5 screens (typically completed in 3-5 minutes in real-world use), as follows: an analysis of the patient’s asthma control and report of patient-entered current medication use (screen 1); guideline-based recommendations for medication adjustments (screen 2); guideline-based recommendations for medications required in the asthma action plan (screen 3); an auto-generated individualized asthma action plan for the patient (screen 4); and a conclusion screen reminding providers to refresh the chart to view an auto-generated chart note documenting CDSS actions, and to create any newly required prescriptions (which are also listed in the chart note).

Screen 1


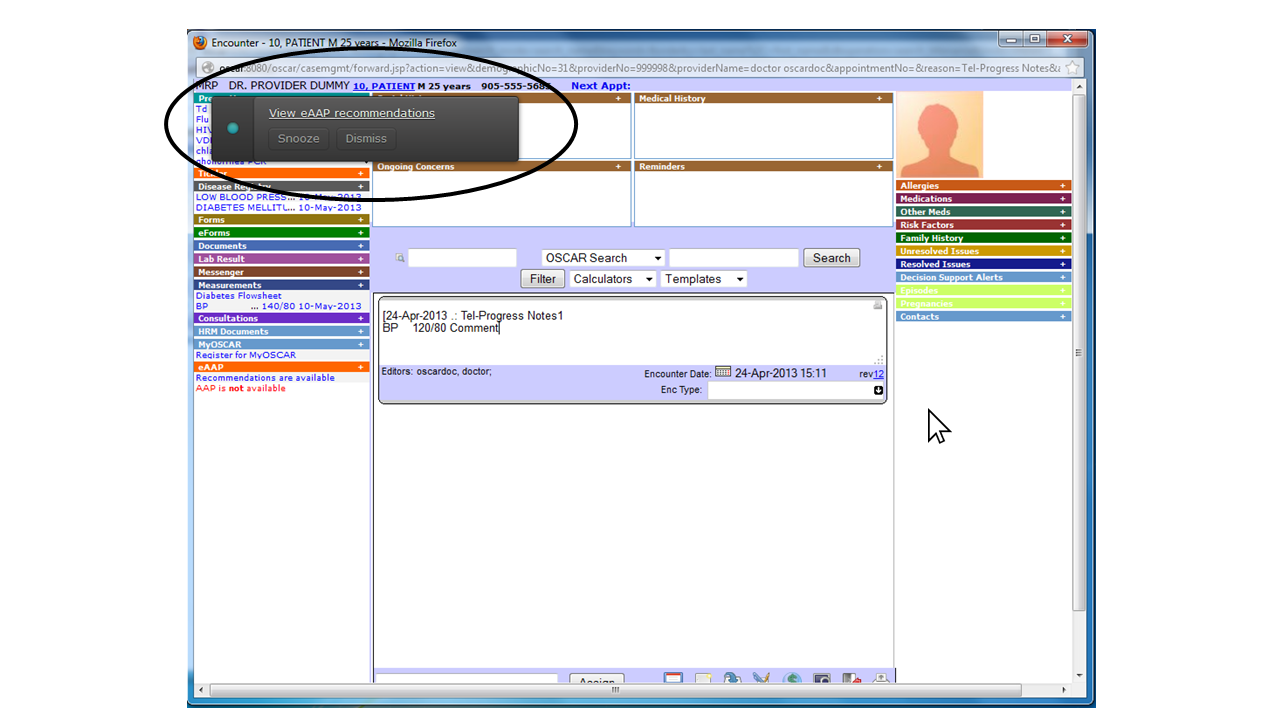

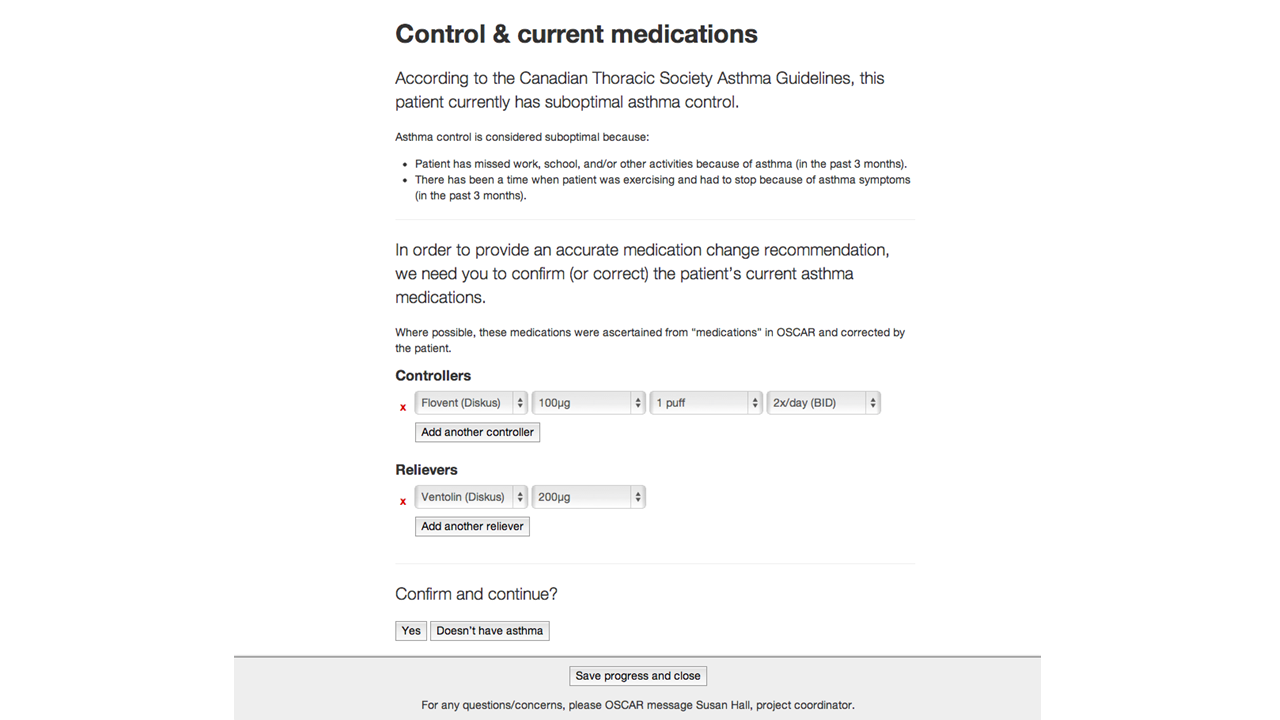


Screen 2


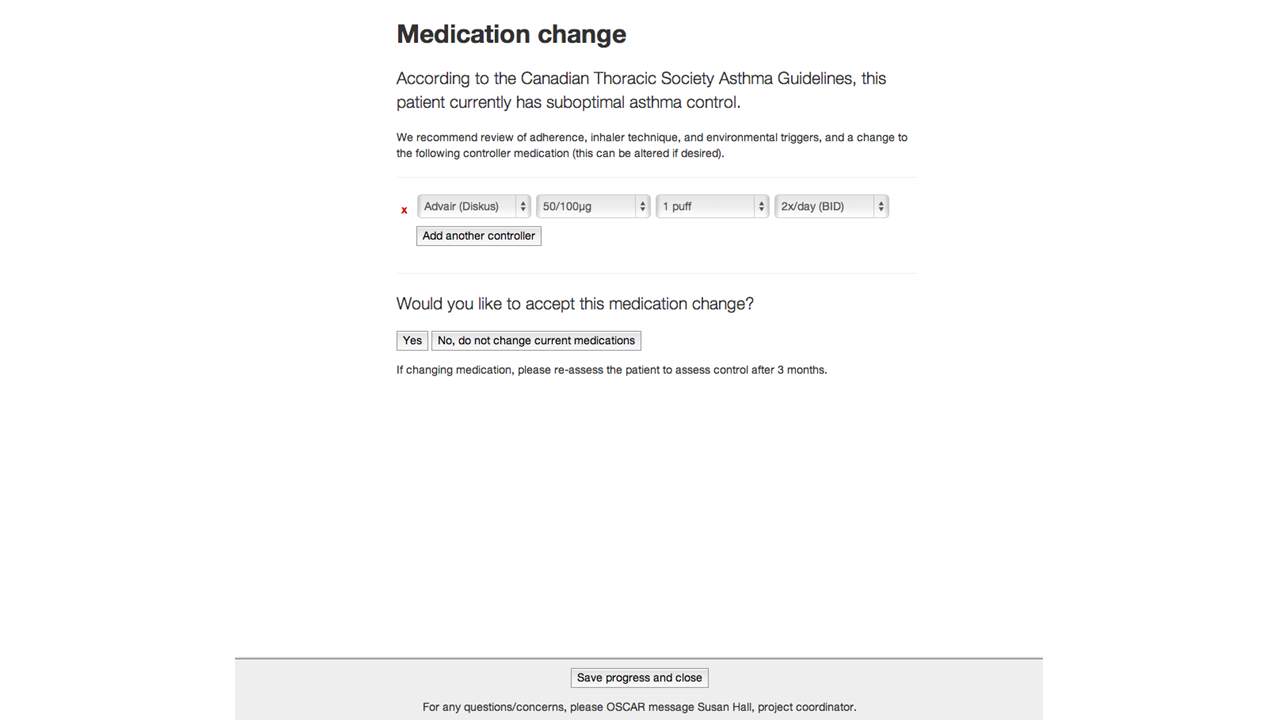


Screen 3


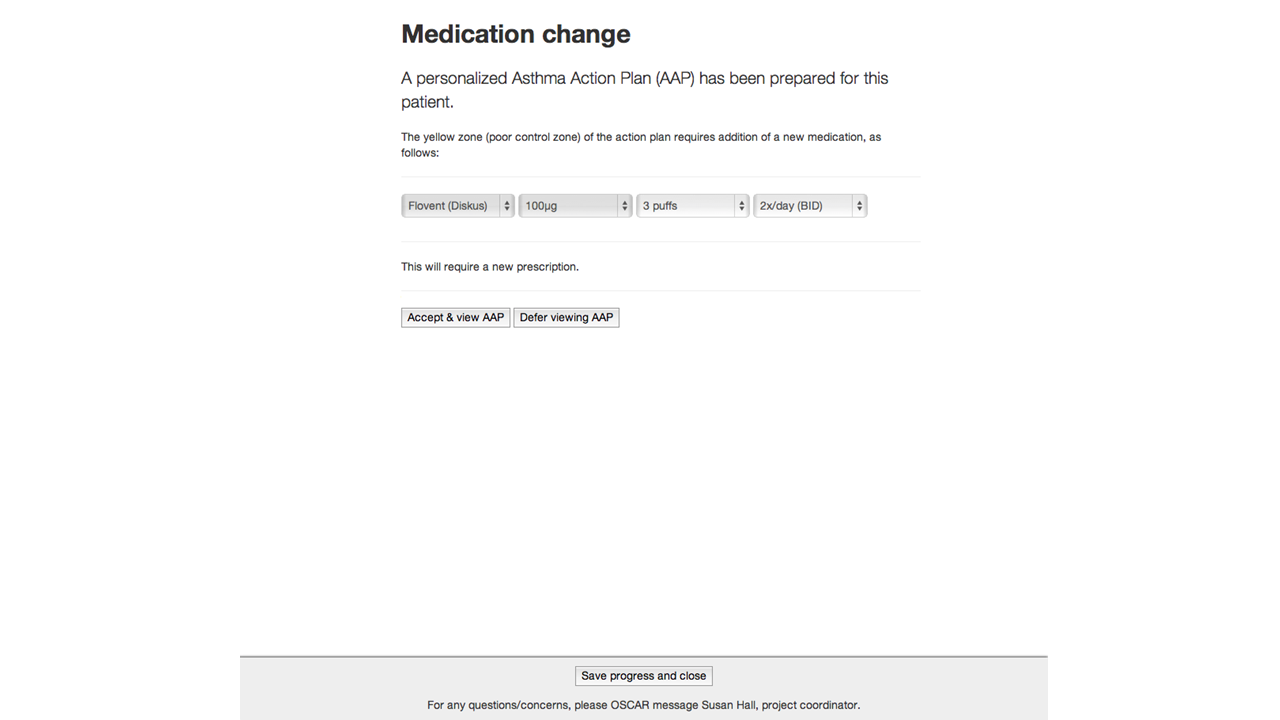


Screen 4


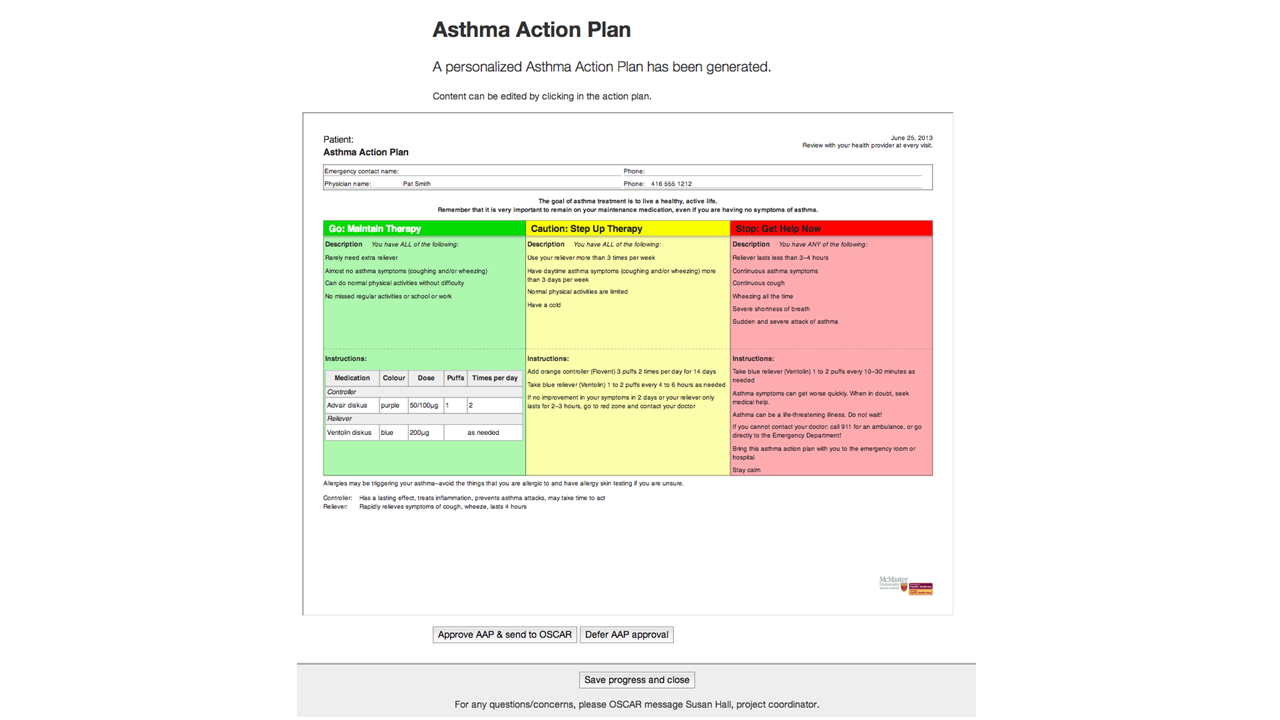


Screen 5


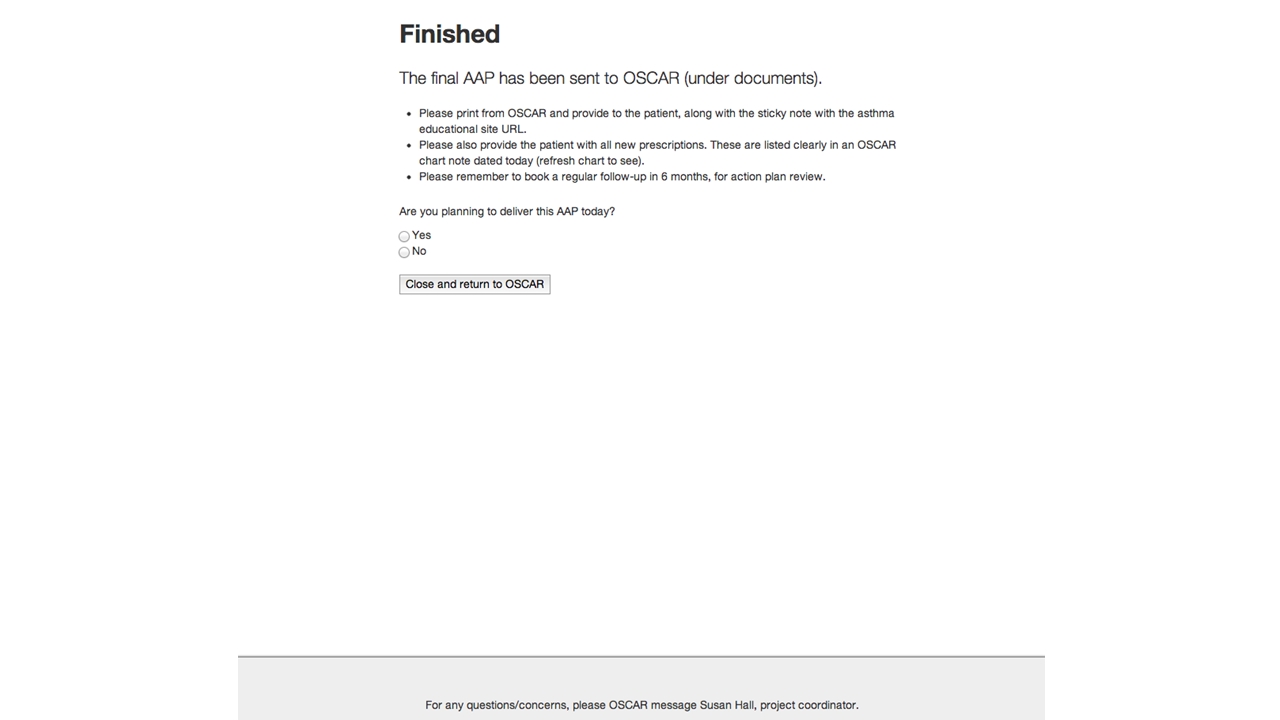

Supplement: Supplementary file 2 — Additional file 2. eAMS Demonstration [file 12911_2023_2273_MOESM2_ESM.docx]
