# Supplementary material for: Improving computerized decision support system interventions: a qualitative study combining the theoretical domains framework with the GUIDES Checklist
Source: BMC Med Inform Decis Mak. 2023 Oct 18;23:226. doi: 10.1186/s12911-023-02273-6 (PMC10585867; doi:10.1186/s12911-023-02273-6)
Supplement: Supplementary file 3 — Additional file 3. Statements Describing Physician Perceptions of Patient Experience [file 12911_2023_2273_MOESM3_ESM.docx]

**Supplementary File 3: Statements Describing Physician Perceptions of Patient Experience**

| **Physician perception (current study)** | | **Patient perception (39)** | |
| --- | --- | --- | --- |
| **Enablers** | | | |
| **Domain** | **Belief Statement** | **Domain** | **Belief Statements** |
| Beliefs about consequences | Using the CDSS enables a more patient centred care approach | Beliefs about Consequences | Completing the questionnaire will facilitate patient centred/personalized care |
| Beliefs about consequences | Completing the CDSS will ensure that I capture all required patient data | Beliefs about Consequences | Completing the questionnaire will provide my doctor with useful information about my asthma control  Completing the questionnaire will help to inform my asthma action plan |
| Beliefs about consequences | Completing the CDSS will improve consistency, transparency and quality of provider patient communication | Goals | Completion of the questionnaire is a priority for me because the quality of my health care will improve |
| Reinforcement | Seeing improved patient outcomes from using the CDSS would influence me to use the system | Beliefs about Consequences | Completing the questionnaire may lead to better asthma management/asthma control |
| Social Influence | I would use the CSS if I received positive feedback from patients | Beliefs about Consequences | Completing the questionnaire will provide my doctor with useful information about my asthma control |
| Social Influence | I would use the CDSS if the patient completes their part | Goals | Completion of the questionnaire is a priority for me because the quality of my health care will improve |
| **Barriers** | | | |
| **Domain** | **Belief Statements** | **Domain** | **Belief Statements** |
| Beliefs about Consequences | Use of a patient questionnaire in the CDSS may weaken the patient-provider relationship |  | Not identified. |
| Beliefs about Consequences | Patient facing CDSS components may create unrealistic patient expectations of care |  | Not identified. |
| Social Influences | Older patients may not have the skills to access patient facing CDSS components and would influence my use of the CDSS |  | Not identified. |

Yamada J, Kouri A, Simard S, Segovia S, Gupta S. Barriers and enablers to using a patient-facing electronic questionnaire: a qualitative theoretical domains framework analysis. J Med Internet Res. 2020;22(10).
